# Resveratrol and p53: How are they involved in CRC plasticity and apoptosis?

**DOI:** 10.1016/j.jare.2024.01.005

**Published:** 2024-01-06

**Authors:** Aranka Brockmueller, Constanze Buhrmann, Amir Reza Moravejolahkami, Mehdi Shakibaei

**Affiliations:** a Chair of Vegetative Anatomy, Institute of Anatomy, Faculty of Medicine, Ludwig-Maximilians-University Munich, Pettenkoferstr. 11, D-80336 Munich, Germany; b Institute of Anatomy and Cell Biology, Faculty of Medicine, University of Augsburg, Augsburg, Germany; c Department of Clinical Nutrition, School of Nutrition & Food Science, Isfahan University of Medical Sciences, Isfahan, Iran

**Keywords:** Colorectal cancer, Resveratrol, P53, Apoptosis, Tumor cell plasticity, Epithelial-mesenchymal transition, Inflammation, Cancer stem cells

## Abstract

**Background:**

Colorectal cancer (CRC), which is mainly caused by epigenetic and lifestyle factors, is very often associated with functional plasticity during its development. In addition, the malignant plasticity of CRC cells underscores one of their survival abilities to functionally adapt to specific stresses, including inflammation, that occur during carcinogenesis. This leads to the generation of various subsets of cancer cells with phenotypic diversity and promotes epithelial-mesenchymal transition (EMT), formation of cancer cell stem cells (CSCs) and metabolic reprogramming. This can enhance cancer cell differentiation and facilitate tumorigenic potential, drug resistance and metastasis.

**Aim of Review:**

The tumor protein p53 acts as one of the central suppressors of carcinogenesis by regulating its target genes, whose proteins are involved in the plasticity of cancer cells, autophagy, cell cycle, apoptosis, DNA repair. The aim of this review is to summarize the latest published research on resveratrol’s effect in the prevention of CRC, its regulatory actions, specifically on the p53 pathway, and its treatment options.

**Key Scientific Concepts of Review:**

Resveratrol, a naturally occurring polyphenol, is a potent inducer of a variety of tumor-controlling. However, the underlying mechanisms linking the p53 signaling pathway to the functional anti-plasticity effect of resveratrol in CRC are still poorly understood. Therefore, this review discusses novel relationships between anti-cellular plasticity/heterogeneity, pro-apoptosis and modulation of tumor protein p53 signaling in CRC oncogenesis, as one of the crucial mechanisms by which resveratrol prevents malignant phenotypic changes leading to cell migration and drug resistance, thus improving the ongoing treatment of CRC.

## Highlights

• Colorectal cancer, which is mainly caused by epigenetic and lifestyle factors, is very often associated with plasticity during its development.
• Tumor cell plasticity leads to metabolic reprogramming of cells, drug resistance metastasis, which is of great therapeutic importance.
• p53 acts as one of suppressors of carcinogenesis by regulating its genes involved in plasticity, autophagy, cell cycle, apoptosis and DNA repair.
• The specific modulation of such proteins by natural compounds as prophylaxis or intervention therefore represents a therapeutic approach.
• There is a link between anti-plasticity and pro-apoptosis by modulating p53 as a key resveratrol anti-CRC mechanism with therapeutic impact.

## Graphical abstract

**Figure:**
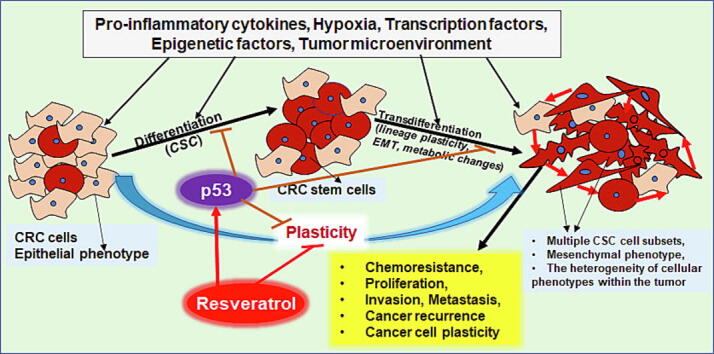

## Introduction

With over 1.9 million new diagnoses annually, colorectal cancer (CRC) affects patients around the world and is particularly common in Europe, Oceania and North America [1]. In addition to the increasing age of the population, this is attributed to a large extent to “modern” lifestyle factors meaning everyday stress, unhealthy diet and lack of exercise [1]. Following a diagnosis of CRC, chemotherapy with drugs such as oxaliplatin and 5-fluorouracil (5-FU) is usually required in conjunction with surgery, which can be a significant physical and psychological burden for the patient.

As an important part of the carcinogenesis, cancer plasticity encompasses a broad spectrum of different interacting cellular phenotypes and behaviors that adapt to each cancer-specific stress, forming a metabolic and functionally heterogeneous group of cells, epithelial-mesenchymal transition (EMT), and cancer stem cells (CSCs), within a tumor [2], [3]. Moreover, it has been reported that such functional cell transformation and plasticity of tumor cell populations are followed by drug-resistance and metastasis, which are ultimately crucial for cancer-related mortality [4], [5], [6]. Therefore, the induction of apoptotic signaling pathways, especially *via* p53, is a fundamental and irreversible protective mechanism to prevent tumor initiation, progression and expansion.

The tumor protein p53, named for its expression at 53 kDa molecular mass, was discovered in 1979 [7], [8] and initially understood to be an oncogene, but the opposite is now known: p53 represents an anti-oncogenic transcription factor that triggers gene expression through deoxyribonucleic acid (DNA) binding and is therefore a tumor suppressor gene [9]. For this reason, the term “cellular tumor antigen p53” has become established too [10] and due to this elementary importance in maintaining a flawless genome as well as significant cancer defense, the nomination “guardian of the genome” was coined [11] electing p53 as “molecule of the year 1993” [12]. Despite its cancer-associated name, p53 is present in all cell types of the body, is continuously expressed and plays a central anti-cancer action by causing cell death, growth arrest as well as senescence and preventing angiogenesis [10], [11], [12]. Through the resulting apoptosis, p53 can exert its effects within the framework of physiological cell differentiation, but also prevent dedifferentiation into a tumor cell during all stages of carcinogenesis or eliminate a degenerated cell [13]. In addition, there is an inverse regulation between p53 and the major inflammatory transcription factor, nuclear factor kappa-B (NF-κB), i.e., phosphorylated pro-inflammatory NF-κB inhibits pro-apoptotic activation of p53 [14] and an induced p53-related apoptosis suppresses the activation of NF-κB [15] in CRC cells. Moreover, a loss of functional p53 in CRC cells is associated with a loss of control over EMT [16] and CSC [17] having a direct effect on cancer cell plasticity.

**Fig. 1:**
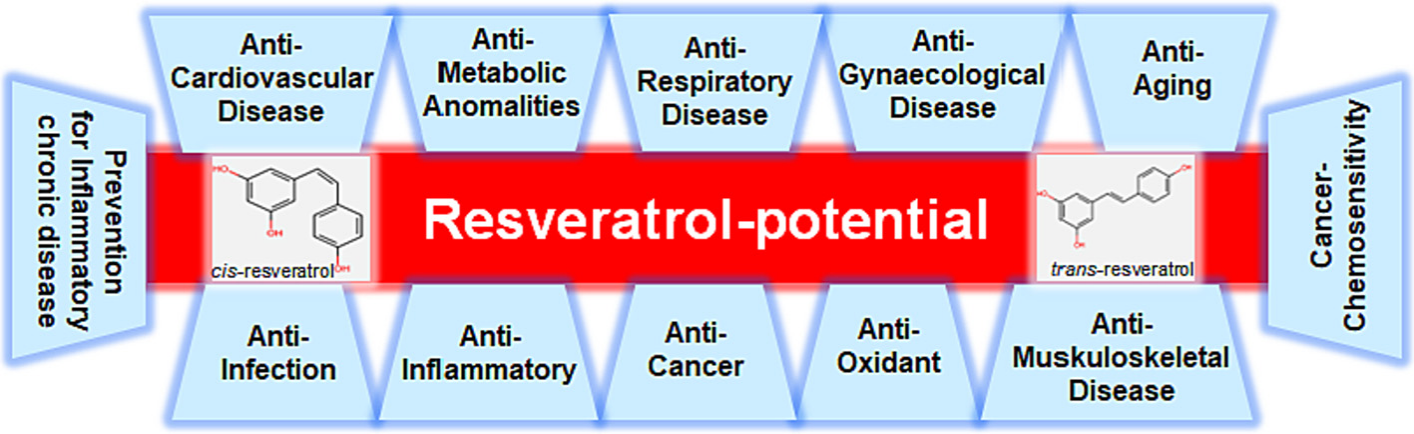
Exemplary health-promoting milestones associated with resveratrol-treatment. The phytopharmaceutical, existing in two isomeric forms, unfolds numerous protective effects in human cells.

To cushion the consequences of multiple disturbed intracellular signals and at the same time to enhance the anti-cancer effect of classical chemotherapeutic agents including the prevention of multidrug resistance or undesirable side effects, phytopharmaceuticals are intensively studied as co-therapeutics [6], [18], [19]. For this purpose, the polyphenol resveratrol, originally arising from numerous healthy foods such as several berries [20], grapes [21], fruit juices and wine [22] as well as nuts [23], represents a forward-looking option. Chemically speaking, resveratrol (3,5,4′-trihydroxy-*trans*-stilbene) is classified as a stilbene and polyphenol with a sum formula of C_14_H_12_O_3_ [24] and two isomeric forms, *cis* and *trans* [25] as shown in Fig. 1. Naturally, it serves plants to defend against aging or pathogens [26] and this function seems to be transferable to consumers of resveratrol sources. In humans, the phytoalexin has proven anti-infective [27], [28], anti-microbial [29] as well as anti-oxidative [30], [31] activities. Thus, resveratrol supplementation protects the cardiovascular [31], [32], metabolic [33], nervous [34], respiratory [35] as well as gynecological and bone [36] system (Fig. 1). Especially, through its successful inflammation control [37], [38], resveratrol can support the prevention and cure of many cancer types including CRC (Fig. 1), where the phytopharmaceutical reduces cell plasticity [6], metastasis and invasion [39], [40] as well as chemoresistance [41], [42]. In addition, resveratrol has been shown to block CSCs in CRC and to induce apoptosis by increasing the expression of p53 and p53-activated Bax (Bcl-2-associated X protein), leading to increased mitochondrial membrane disruption and a release of cytochrome *C* as well as caspase-3 [43].

In this context, this review describes a novel link between anti-functional plasticity (EMT, CSCs, metabolism), pro-apoptosis and modulation of p53 signaling pathway in CRC cell oncogenesis as one of resveratrol’s key mechanisms to suppress or reverse malignant phenotype, cell migration ability and resistance to conventional drugs *in vitro* and *in vivo*, suggesting a major therapeutic significance.

## Fundamentals of this research

### Focus of this review

In this review, we present known facts about tumor protein p53, the natural polyphenol resveratrol as well as CRC analyzing their overlaps and interrelationships with regard to inhibition of functional malignant cell plasticity and apoptosis initiation in CRC cells. Overall, we explore the question of whether resveratrol modulates the p53 signaling pathway as functional anti-plasticity, anti-migration and pro-apoptotic key in CRC cells.

### Data collection of this review

The PubMed database served as the source for the data summarized here, using the following keywords in various topic-specific combinations: “CRC”, “colorectal cancer”, “resveratrol”, “p53”, “tumor suppressor protein”, “apoptosis”, “inflammation”, “EMT”, “CSCs”, “cancer stem cells”, “stemness”, “plasticity”, “tumor cell plasticity”.

## Pathogenesis of CRC

Cancer is one of the most prevalent diseases and due to its constant risk of recurrence, it is considered chronic. Ten percent of annual 19.3 million new cancer cases worldwide suffer from CRC and almost 1 million new cancer deaths are associated with it every year [1]. The pathogenesis of CRC usually follows three stages: initiation, promotion as well as progression. During initiation, DNA is damaged by toxic, genetic or epigenetic influences and this damage is aggravated during promotion by accumulation of preneoplastic cells. The malignant expansion, which is associated with high EMT, CSC growth rates, phenotypic plasticity, invasiveness and metastasis, is referred to as progression [44]. Thereby, CRC carcinogenesis is not only genetically variable but can also be triggered by various individual factors. In this regard, due to its highly varied profile of gene expression, a division into hypermutated or non-hypermutated types is made [45].

CRC begins when epithelial cells in the colon or rectum are severely damaged and altered by genetic or epigenetic mutations such as deviations in DNA methylation, histone modifications, chromosome restructuring with chromosomal instability (CIN) or microsatellite instability (MSI), and violation of the mismatch repair (MMR) system [45]. When the MMR system is disrupted, the genome becomes unstable, as it is important for proofreading DNA synthesis errors during replication. This leads to an altered functional cell phenotype and metabolic plasticity, increased susceptibility to neoplastic cell transformation and favors the development of chemoresistant cells [46], [47]. As a result, initially formed, benign adenomas become serrative neoplasms, which can develop into metastatic carcinomas [48], [49]. In CRC, the genetic changes are mostly mutations in p53 [50], KRAS, NRAS or BRAF, where KRAS mutations are associated with a poor prognosis in particular because they go along with persistent or recurring liver metastases [51]. Only 5–10 % of CRC patients suffer from a hereditary form such as familial adenomatous polyposis (FAP) or hereditary nonpolyposis CRC (HNPCC). However, these variants are associated with a high lifetime risk of 80–100 % [52]. Additionally, recent evidence shows, that inflammatory processes in the intestine, such as inflammatory bowel diseases, can cause healthy cells in the gut epithelium to develop dysplasia to varying degrees, which ultimately play an important role in the development of CRC after a long time [53]. Overall, in all stages of CRC development, concretely initiation, promotion as well as progression, a balanced relationship of p53 plays a crucial role, because this tumor protein is directly or indirectly involved in all plasticity-associated processes [16], [17].

At CRC diagnosis, the median age is between 63 years (rectal) and 69 years (colon) and the rate of incidence is increasing with rising patient age but in recent years, increasing numbers of cases have been observed among younger people [54]. Most commonly, CRC is located in proximal colon (40 %), followed by rectum (29 %), distal colon (22 %) and others (9 %) [54].

Various medications are available for CRC treatment, which are usually used after a primary operation and from stage II, depending on the patient case. These include, to mention a few, 5-fluorouracil (5-FU), cisplatin, oxaliplatin and irinotecan [18]. Furthermore, the age of individual therapy has recently begun and since then protein kinase inhibitors such as encorafenib or monoclonal antibodies such as pembrolizumab, cetuximab, pertuzumab and trastuzumab have also been used [55]. All these synthetic chemotherapeutics can cause therapy-limiting side effects such as nausea, vomiting, weakness, toxicity or lead to the dreaded chemoresistance. Nevertheless, the mortality as well as recurrence rate in patients with CRC continues to be quite elevated [18]. Therefore, and because it takes many years for dysplasias to develop into cancer, the use of prophylactic agents or co-treatments with few side effects and with the focus on inducing early death of degenerated cells is being highly researched. Phytopharmaceuticals such as resveratrol have an advantage here, especially because of their multifunctional ability as a therapeutic and chemopreventive agent used in the treatment of a variety of diseases, including cancer [6], [56].

## Formation of CRC plasticity

The plasticity of a cell represents a physiological und necessary characteristic during embryonic differentiation by which stem cells can develop into terminal cells of a human body. The phosphoprotein p53 as well as zinc finger transcription factors ‚Snail Family Transcriptional Repressor 1‘ (Snail) and ‚Snail Family Transcriptional Repressor 2‘ (Slug) are considered as main regulators of embryogenesis and organogenesis [57], [58]. In this relation, EMT takes a central role causing a loss of cell polarity, thus enabling the cells to acquire a functional phenotypical change from resident-epithelial to migratory-mesenchymal [59].

**Fig. 2:**
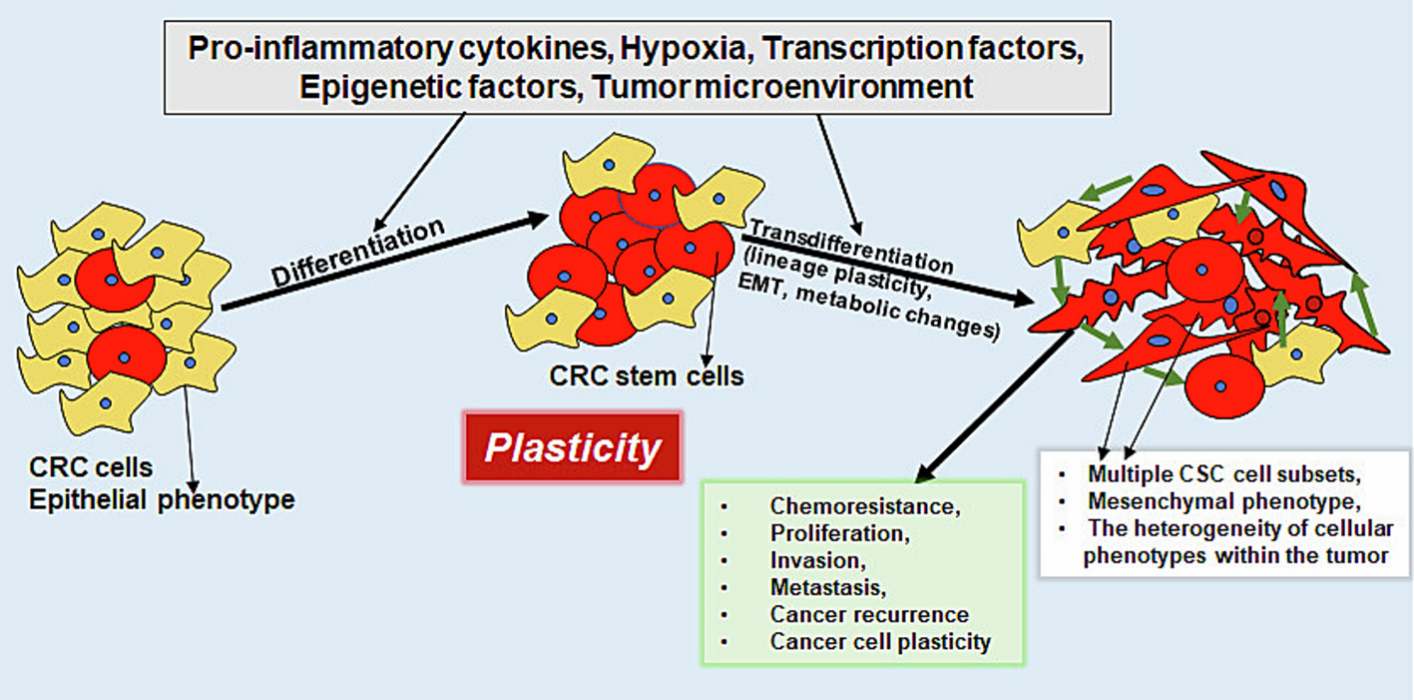
Tumor cell phenotypic plasticity in CRC cells. Tumorigenesis-associated stress factors lead to the development of CRC and promote the differentiation as well as transdifferentiation of CRC cells (black arrows). As a consequence, plasticity changes (violet arrow) are induced resulting in a challenging course of the disease. Abbreviations: EMT - epithelial-mesenchymal transition, CRC – colorectal cancer, CSC – cancer stem cell.

However, these prerequisites and abilities described for cell movement are also associated with malign tumors in the course during life. The current state of research assumes that A) pathological cancer initiation is triggered by inflammatory processes [60], B) malign tumors arise from CSCs [61] and further, that C) cancer cells are able to reactivate the EMT-process [62] to metastasize (Fig. 2).

All three phenomena were frequently observed in CRC tumorigenesis and go along with enormous phenotypic and functional heterogeneity, up to transformation from benign into malign cell types. Especially related to CRC development, an adenoma-carcinoma sequence caused by neoplastic transdifferentiation of colonic epithelium is often involved [63]. Furthermore, an intensive intercellular crosstalk between CRC cells and numerous other cell types as well as paracrine signals leads to a spread of inflammation, an up-regulation of CSCs and a promotion of EMT [64]. The resulting mesenchymal CRC cells are characterized by multiple plasticity-related changes such as an increased β1-integrin receptor expression [65] and development of pro-migratory cell membrane pseudopodia [41].

Due to the significant consequences of metastasis-promoting plasticity-mechanisms and its pro-apoptotic counter-regulation, a special attention is paid to resveratrol’s p53 modulatory function on intracellular phenotype changes in CRC.

## Resveratrol suppresses CRC cell plasticity *via* p53 signaling

### Modulation of inflammation-induced CRC cell plasticity by resveratrol

The inflammation-induced release of cytokines, chemokines, enzymes and pro-inflammatory transcription factors such as NF-κB creates an environment that promotes the development of chronic diseases such as cancer. A typical example is bowel cancer, which is favored by chronic inflammatory bowel diseases [66] and is also based on a specific tumor microenvironment (TME) that develops into a self-reinforcing spiral through a lively crosstalk between stromal cells, immune cells, intestinal cells and arisen CRC cells [67]. The cascades that are subsequently triggered create optimal conditions for the growth of CRC cells and lead to phenotypic changes that facilitate migration [67]. In addition, a microbiome out of sync due to inflammatory processes forces the CRC stemness including proliferation and invasion behaviour of the tumor cells [68], because of which an inflammation-induced plasticity of CRC cells is therefore indisputable. Especially against the background of the well-known adenoma-carcinoma sequence in CRC, cellular aging is also an additional inflammatory accelerator, in the process of which p53 is significantly involved. Overall, it has been reported that in the relationship between inflammation and cancer cell plasticity, inflammation controlled by activated NF-κB should affect natural tumor suppressor proteins, such as p53 [69].

The phytopharmaceutical resveratrol is known as a potent anti-inflammatory agent, especially in CRC cells, where it modulates multiple inflows, activation mechanisms and consequences of inflammation’s main switch, particularly the transcription factor NF-κB, which represents one of the major pro-inflammatory transcription factors and regulates over 500 different genes [70]. Interestingly, the natural substance up-regulates p53 including its post-translational modification in consistence with its pro-oxidant action [71], and down-regulates the enzymatic activity of cyclooxygenase (COX)-2 [72] as well as the liberation of cytokines such as tumor necrosis factor (TNF)-α, TNF-β [73] or interleukin (IL)-6 [74] that are related to phenotypic and metabolic plasticity in the CRC-TME (Table 1).

**Table 1:** Prevention of CRC cell plasticity through resveratrol treatment *in vitro*.

**CRC cell lines**	**Resveratrol treatment**	**Inhibition of**		**Targets of resveratrol**	**Year**	**Reference**
		**CSC**	**EMT**			
LoVo	6–200 µM, 48–72 h		x	From 6 µM on, resveratrol suppressed EMT (E-cadherin, Snail, vimentin) *via* targeting TGF-β1/Smad signaling.	2015	[78]
HCT-116	1–50 µM, 10–22 days		x	From 5 µM on, resveratrol impeded NF-κB activation, invasion (MMP-9) and enhanced apoptosis (caspase-3) resulting in EMT (E-cadherin, Slug, vimentin) control.	2015	[84]
CSCs	9 µM, 24 h	x		At 9 µM, resveratrol (in combination with grape seed extract) modulated Bax:Bcl-2 ratio, PARP, c-Myc, cyclin D1, p53 and Wnt/β-catenin pathway.	2016	[43]
HCT-116	25–400 µM, 24–28 h		x	At 30 µM, resveratrol prevented EMT by decreasing of ZEB-1 and vimentin expression as well as increasing E-cadherin and miR-200c expression.	2017	[82]
HCT-116	5 µM, 10 days	x	x	At 5 µM, resveratrol reduced CSC-related (CD44, CD133, ALDH1), inflammatory (NF-κB) and proliferative (CXCR4, MMP-9) and mesenchymal (vimentin, Slug) markers, while it promoted epithelial (E-cadherin) and apoptotic (caspase-3) parameters.	2018	[73]
HCT-116, RKO, SW480	5 µM, 14 days	x	x	At 5 µM, resveratrol repressed inflammation (NF-κB), CRC progression (FAK, Ki-67, MMP-9, CXCR4) and CSC production (CD44, CD133, ALDH1). Moreover, it prevented EMT by balancing vimentin, Slug and E-cadherin.	2019	[83]
SW480, SW620	3.75–240 µM, 48 h		x	At 15 µM, resveratrol modulated EMT by down-regulation of Snail and up-regulation of E-cadherin. Additionally, it inhibited the phosphorylation of Akt and GSK‑3β.	2019	[85], [91]
HCT-116	1–10 µM, 14 days	x		From 5 µM on, resveratrol suppressed CSC development (ALDH1, CD44, CD133), inflammation (NF-κB) and proliferation (Ki-67, MMP-9, CXCR4). In parallel, it promoted apoptosis (caspase-3) and Sirt-1 regulation.	2020	[64]
LoVo, LoVoDX	1–5 µM, 24 h	x		At 5 µM, resveratrol inhibited CSC formation by up-regulation of Sirt-1/-2/-3/-6, reduction of ROS and BRCA1/PARP1 modulation.	2022	[89]
HCT-116	1–5 µM, 10–14 days	x	x	At 5 µM, resveratrol prevented EMT-switch by suppression of inflammation NF-κB and reduced CSC production by down-regulation of ALDH-1, CD44 and CD133.	2023	[41]

Moreover, resveratrol treatment prevents inflammatory and plasticity-activating reactive oxygen species (ROS) and p53 deacetylation associated with colon cancer transformation, proliferation and metastasis [72]. In a further step, the grape-derived polyphenol influences directly the NF-κB expansion by suppression of its gene expression resulting in an inhibition of inflammation activation and an interruption of NF-κB inflammatory and cancerogenic end product formation. This becomes tangible in different CRC cell lines, HCT-116 and RKO for example, through an inhibition of migratory markers such as matrix metalloproteinase (MMP)-9 as well as CXC motif chemokine receptor (CXCR)4 and at the same time an up-regulation of p53 and caspase-3 activation is thereby made possible [65], necessary for plasticity-containment and apoptosis-initiation [75]. Resveratrol’s intracellular signal transmission is not yet clarified in detail, but β1-integrin receptors are suggested to be involved in its anti-inflammatory, viability-inhibiting and plasticity-suppressing effects in HCT-116, RKO and SW480 CRC cells [39], [65]. Complementing these findings, it has been demonstrated that the phytopharmaceutical is able to suppress an inflammatory T-cell reaction by modulating the CRC-associated p53 pathway [76], which has a significant positive impact on the gut microbiome and thereby enhances 5-year survival of patients with CRC [77]. Altogether, resveratrol represents an important natural NF-κB and thus inflammation inhibitor, as it is able to simultaneously activate the tumor protein p53 and p53-associated downstream target genes, which can lead to inhibition of cell plasticity [14], a fact that is particularly relevant in CRC therapy.

### Modulation of EMT-induced CRC cell plasticity by resveratrol

Environmental stress factors and inflammation with chemokines, cytokines and activated NF-κB induce an EMT-derived mesenchymal phenotype and changes in related expression parameters are involved in cell plasticity. While EMT causes a decrease in epithelial, transmembrane glycoprotein E-cadherin, it causes an increase in mesenchymal, filamentary protein vimentin as well as Snail, Slug and regulatory adhesion adapter protein paxillin, which is also an EMT-associated tumor cell plasticity marker [39], [78], [79].

As p53 regulates several processes of differentiation, the mitigation of its oncosuppression is also discussed as pro-carcinogenic and EMT-inducing factor in a tumor development [62]. For example, the already mentioned inflammatory activities *via* NF-κB pathway suppress an activation of p53 and thus apoptosis induction in cancer cells [80]. Otherwise, a functioning p53-cascade is known to counteract the mesenchymal plasticity of CRC cells [81].

The phytopharmaceutical resveratrol represents also a significant inhibitor of EMT processes in CRC cells and is even able to reverse a mesenchymal-altered phenotype into a more epithelial phenotype, called mesenchymal-epithelial transition (MET), in the most investigated HCT-116 cell line, for instance [82]. Moreover, these observations were reproduced in further CRC cell lines (HCT-116, RKO, SW480) and interestingly, resveratrol interrupts inflammation-related cell plasticity changes to impede the migration and invasion of CRC cells [83]. Impressively, a treatment with the phytopharmaceutical causes a concentration-dependent inhibition of tumor marker paxillin and simultaneously an enhanced E-cadherin-containing plaque deposition in HCT-116 and RKO cells [39]. At the ultrastructural level, a repression of CRC cells‘ metastasis-related pseudopodia became visible, which has been turned into a smooth, epithelial surface by resveratrol addition [41]. The suppression of plasticity-promoting NF-κB as well as focal adhesion kinase (FAK), which both are intertwined with p53 signaling, serves as specific targets for this [83]. In addition, by down-regulation of NF-κB including its end products such as MMP-9, and up-regulation of p53 and intercellular junctions in parallel, resveratrol inhibits not only EMT but also enhances CRC cells‘ sensitivity to the standard chemotherapeutic drug 5-FU [84] (Table 1).

Furthermore, resveratrol demonstrated a concentration-dependent reduction in migration as well as invasion capacities in LoVo cells by blocking the TGF-β1/Smad pathway, inhibiting vimentin and snail expression and up-regulation of E-cadherin [78]. In SW480 and SW620, two further human CRC cell lines, a treatment with the natural polyphenol caused an enhanced E-cadherin, while N-cadherin, snail and GSK‑3β were down-regulated. In this study, the AKT1 kinase was suggested as key regulator of resveratrol’s EMT-inhibition [85] and it is known that their signaling is interconnected with the p53 signaling pathway (Table 1). A recent study complements these findings by demonstrating resveratrol’s p53-dependent prevention of EMT, CRC plasticity and migrating behaviour by phase contrast as well as on ultrastructural level [75]. Overall, this underscores the central role of p53 in CRC cell plasticity suppression while emphasizing the broad potential of modulation of its signaling cascade by resveratrol.

### Modulation of CSC-induced CRC cell plasticity by resveratrol

CSCs are defined as a functional sub-group of resistant tumor cells that possess the property of self-renewal, whereby their exact origin and mechanisms have not yet been clarified but they have been found in large numbers in CRC [86]. Interestingly, a loss of functional tumor protein p53 correlates with an increasing pool of functional CSCs plasticity in CRC leading to a tumor growth promotion, proliferation and invasion [87]. Detailed investigations identified cluster of differentiation (CD)44, CD133 as well as aldehyde dehydrogenase (ALDH)1 as specific CSC markers and demonstrated that their expression is attached to an increased CSC plasticity and decreased apoptosis induction in both, sporadic CRC and also ulcerative colitis related CRC [88].

Moreover, CSCs are crucial for the development of chemoresistance to standard drug therapy and thus of associated phenotypic and metabolic plasticity as well as metastatic cells. Interestingly, the grape constituent resveratrol has been shown to have a significant anti-carcinogenic effect on both CRC cells and CSCs in the same colorectal tumors [89]. In this regard, as shown in Table 1, a limitation of CSCs plasticizing in HCT-116, RKO and SW480 CRC cells was proven repeatedly by resveratrol’s down-regulation of CD44, CD133 and ALDH1 parameters [64], [73], [83]. Furthermore, *via* using β1-integrin/hypoxia-inducible factor (HIF)-1α pathway, a treatment with resveratrol suppresses inflammation, neoangiogenesis as well as expression of CSC markers. In parallel, a progress of apoptosis and even sensibility against chemotherapeutic agent 5-FU is promoted, so that resveratrol exerts a strong overall anti-CRC effect in essential parts through cancer cell plasticity inhibition [6], [41].

Complementing these *in vitro* studies, a combined *in vitro/in vivo* examination showed an inhibition of Wnt/β-catenin pathway and an increase in mitochondria-related programmed cell death *via* p53 signaling after resveratrol treatment in human colon CSCs [43]. Interestingly, Shen and his colleagues demonstrated that the polyphenol resveratrol clearly interferes with the p53 signaling pathway to inhibit EMT, CSCs and their metabolic reprogramming and phenotypic plasticity in nasopharyngeal carcinoma CSCs [90]. Collectively, resveratrol serves as a potent inhibitor of CSC formation and EMT-associated phenotypic and metabolic plasticity of CRC cells, as outlined in Table 1.

### Modulation of p38/MAPK-related CRC cell plasticity and apoptosis by resveratrol

**Fig. 3:**
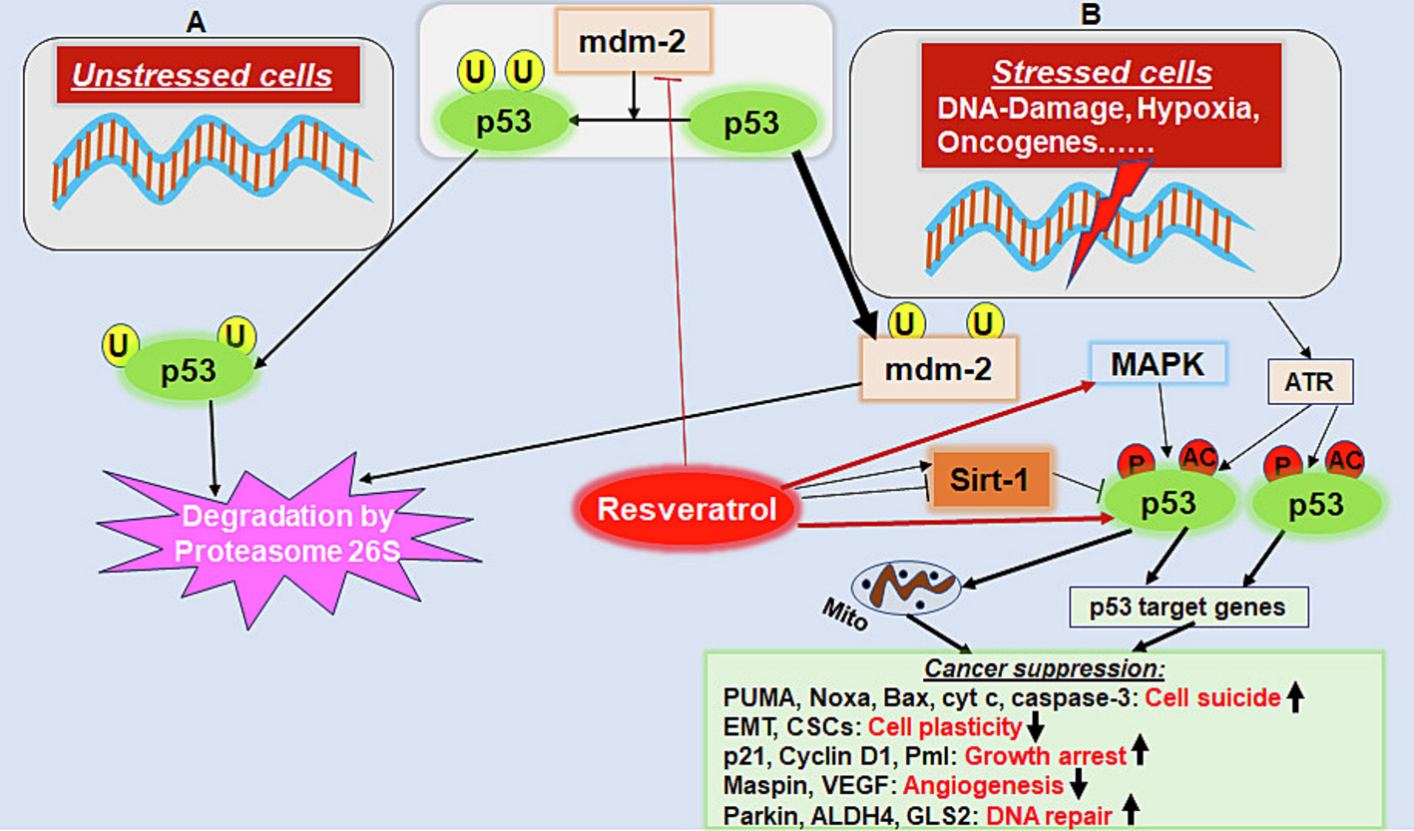
Representation of the anti-cancer action of resveratrol by induction of apoptosis and inhibition of cellular plasticity in cancer cells, mediated by the protein p53. (A) In unstressed cells, the inhibition and periodically degradation of p53 is controlled by mdm-2 oncogene. (B) In cells stressed by environmental factors, p53 is activated leading to programmed cell death and cancer suppression (green background). Resveratrol (red background) is able to influence the status of cancer cells *via* Sirt-1, MAPK or p53 pathway amplifying the anti-CRC effect of p53. Abbreviations: ATR - Ataxia Telangiectasia and Rad3, ALDH - aldehyde dehydrogenase, Bax - Bcl-2-associated X protein, CSCs – cancer stem cells, cyt c – cytochrome *C*, DNA - deoxyribonucleic acid, EMT - epithelial-mesenchymal transition, GLS2 - phosphate-activated mitochondrial glutaminase, MAPK - mitogen-activated protein kinase, mdm-2 - murine double minute 2 gene, Noxa - phorbol-12-myristate-13-acetate-induced protein 1, Pml - promyelocytic leukemia gene product, PUMA - p53 upregulated modulator of apoptosis, Sirt – sirtuin.

A controlled, signaling pathway-driven programmed cell death that does not trigger a subsequent inflammatory spread is referred to as apoptosis. It represents a basic prerequisite for the proper development of an organism, but also serves to remove harmful degenerated cells which is particularly important with cancer cells [92]. The family of MAPKs (mitogen-activated protein kinase) summarizes versatile and exceptional enzymes, their activation requires serine-, threonine- as well as tyrosine-phosphorylation [93]. Different members of MAPK family are involved in molecular processes of differentiation, inflammation, tumorigenesis or apoptosis, underlining their relevance in cancer diseases. One of the decisive representatives in CRC is p38/MAPK, which seems to play a crucial role in enabling apoptosis here [94]. Due to carcinogenesis-related stress factors such as an increased amount of cytokines in the TME, the p38/MAPK signaling is activated, resulting in an inhibition of CRC cell viability and metastatic possibilities by stimulation of p53. Furthermore and in parallel, the induction of programmed cell death is promoted by the pathway switch [95]. In this context, a p38-initiated autophagy is known that correlates with a repression of mammalian target of rapamycin (mTOR) pathway as well as a mitochondria-mediated apoptosis in CRC [96], for example in HT-29 or Caco-2 cells. More precisely, the extensive signaling cascades about caspase-3, −8 and −9 [97] are activated in the following. Another known mechanism triggered by p38/MAPK phosphorylation directly influences the cell cycle of CRC cells through inhibition of cyclin D1/cyclin-dependent kinase (CDK)4 complex responsible for G1/S-phase transition and beyond that there are intertwined connections with the inflammatory NF-κB pathway [98]. Overall, an initiation of p38/MAPK cascade unfolds numerous pro-apoptotic options in CRC cells. Of great interest is particularly the finding that the activation of p38, for instance in SW480 or SW620 cells, entails the initiation of PUMA (p53 upregulated modulator of apoptosis)/p53 signaling pathway [99] (Fig. 3), so that this signaling can be viewed as part or prelude to this main apoptosis cascade as well as an interesting molecular target to suppress CRC progression.

The natural polyphenol resveratrol showed versatile anti-proliferative [65], [100] as well as anti-plasticity and anti-metastatic [39], [40] effects in different CRC cell lines and numerous signaling pathways are involved making it a poly-target molecule. Considering its pro-apoptotic properties (Table 2), a resveratrol treatment directly activates the cascades of caspase-3/-6/-8/-9 [84], [101], [102], [103] and thus apoptosis in CRC cells *via* up-regulation of ROS [104], nitric oxide (NO), p53 signaling [102], modulation of Bax/Bcl-2 homologous antagonist/killer (Bak) ratio [102], [105], [106] or p38/MAPK pathway [107], [108], [109], [110].

**Table 2:** Apoptotic pathways in CRC cells targeted by resveratrol *in vitro*.

**CRC cell lines**	**Resveratrol treatment**	**Signaling pathway**	**Modulation by resveratrol**	**Year**	**Reference**
HCT-116	200 µM, 5–12 h	Caspase-6	Up-regulation of caspase-6 and thus induction of lamin A cleavage was identified as an important pathway for the initiation of apoptosis by resveratrol.	2006	[103]
HCT-116	100 µM, 24–72 h	Bax/Bak	Promotion of pro-apoptotic proteins Bax and Bak by resveratrol was crucial for its chemopreventive effects.	2006	[106]
HCT-116, Caco-2	30–200 µM, 24–72 h	Sirt-1, PPAR-γ, p38, MAPK	Modulation of proliferation-associated metabolism by resveratrol *via* increasing Sirt-1, PPAR-γ and p38/MAPK pathway.	2006	[109]
HCT-116	25–1600 µM, 24 h	Caspase-6	Initiation of apoptosis using caspase-6 signaling was promoted by resveratrol alone or in combination with 5-FU.	2008	[101]
HT-29	10–400 µM, 6–72 h	ROS	Activation of ROS- and caspase-3-dependent apoptosis by resveratrol inhibited cell proliferation without sign of cytotoxicity.	2008	[104]
HCT-116	10–50 µM, 5–21 days	NF-κB	Inhibition of NF-κB pathway by resveratrol alone or in combination with curcumin attenuated proliferation and stimulated apoptosis.	2009	[113]
HCT-116	10–200 µM, 6–48 h	NO, p53, Bax, caspase-8/-9	Up-regulation of NO, p53, Bax and caspase-8/-9 crystallized as central strategies of resveratrol-induced apoptosis.	2009	[102]
HCT-116	50–200 µM, 24 h	Caspase-6, p53	Promotion of caspase-6 and p53 by resveratrol induced apoptosis and acted synergistically with 5-FU.	2009	[118]
HT-29, SW480	50–150 µM, 24–72 h	IGF-1R/Akt/Wnt, p53	Suppression of IGF-1R/Akt/Wnt signaling and up-regulation of p53 by resveratrol, thereby inhibition of proliferation and initiation of apoptosis.	2010	[115]
HCT-116	10–60 µM, 6–72 h	JNK, p38, MAPK	Inhibition of cell cycle at S-phase and initiation of DNA-damage-associated apoptosis *via* p38/MAPK signaling by resveratrol. Further, a synergistic effect of 5-FU and resveratrol was observed.	2011	[107]
HT-29	50–400 µM, 24–72 h	PKC-ERK1/2	Initiation of apoptosis and reduction of proliferation by resveratrol through regulation of PKC-ERK1/2 pathway.	2012	[119]
HCT-116	20–100 µM, 24–72 h	PTEN/PI3K/Akt, Wnt/β-catenin	Modulation of PTEN/PI3K/Akt and Wnt/β-catenin signaling, thereby enfolding of anti-proliferative as well as pro-apoptotic effects by resveratrol.	2014	[114]
HT-29	12.5–50 µM, 24 h	IFN-γ, IL-1α, TNF-α, NO, PGE2, iNOS, COX-2, JAK/STAT, p38/MAPK	Down-regulation of JAK-STAT and simultaneously up-regulation of p38/MAPK pathway, through which resveratrol limited inflammation and promoted remission.	2014	[108]
HCT-116, SW480	1–50 µM, 10–22 days	NF-κB, MMP-9, caspase-3	Down-regulation of NF-κB, MMP-9 and up-regulation of caspase-3 by resveratrol acted anti-inflammatory as well as pro-apoptotic. Moreover, resveratrol prevented EMT and chemosensitized to 5-FU.	2015	[84]
HCT-116	0.0001–100 µM, 72 h	Bax	Bax up-regulation and chemosensitization to doxorubicin by resveratrol led to activation of apoptosis.	2016	[105]
LoVo	10–80 µM, 24–48 h	BMP9, MAPK, p38	Activation of p38/MAPK signaling cristallized as a central anti-proliferative mechanism of resveratrol.	2016	[110]
HCT-116, SW480	5 µM, 10–28 days	FAK	Inhibition of FAK signaling pathway and thereby attenuation of invasion by resveratrol.	2017	[116]
HCT-116, HT-29	10 µM, 24–96 h	COX-2, p53	COX-2 down-regulation and p53 up-regulation by resveratrol led to inflammation-inhibition as well as limitation of proliferation.	2018	[111], [112]
HCT-116, SW480, RKO	1–20 µM, 10–14 days	β1-integrin, NF-κB	Suppression of NF-κB using β1-integrin receptors for transduction of anti-viability, anti-proliferative, anti-metastatic and pro-apoptotic signals by resveratrol.	2022	[39], [65]
HCT-116	10–80 µM, 24–72 h	Hippo/YAP	Activation of Hippo/YAP pathway was initiated by resveratrol to suppress proliferation and induce apoptosis.	2022	[117]
HCT-116	1–5 µM, 10–14 days	β1-integrin, HIF-1α	Suppression of HIF-1α using β1-integrin receptors through resveratrol, thereby inhibition of inflammation, vascularization and CSC formation. In addition, resveratrol promoted apoptosis in synergism with 5-FU.	2023	[41]

Aside from that, a treatment with resveratrol leads to an increased expression of bone morphogenetic protein 9 (BMP9) and subsequently to a CRC growth attenuation with apoptosis induction *via* up-regulation of p53-related p38/MAPK pathway [110] in LoVo cells. Moreover, Sirt-1 and peroxisomal proliferator-activated receptor gamma (PPAR-γ) were identified as resveratrol’s mediators to enhance p38/MAPK in HCT-116 and Caco-2 CRC cells [109]. The activation of p38/MAPK signaling as one of resveratrol’s essential targets was repeatedly reproduced in HCT-116 cells (Table 2). Here, a cell cycle arrest at S-phase as well as a significant enhancement of CRC cell death induction was observed and furthermore, the anti-cancer effects of 5-FU were improved when the phytopharmaceutical was used as co-treatment to the classic chemotherapeutic agent [107]. Also in HT-29 cells, an addition of resveratrol causes an inhibition of janus kinase (JAK)/signal transducers and activators of transcription (STAT) signaling with a simultaneous promotion of pro-apoptotic p38/MAPK pathway (Table 2) and additionally, a striking suppression of numerous inflammatory processes was noticeable [108].

Moreover, resveratrol is able to block pro-inflammatory pathways in CRC cells at several steps, mainly by its down-regulation of inflammation-triggered COX-2 [111], [112] and further, the natural polyphenol has a particularly strong effect as natural inhibitor of NF-κB. The repression of NF-κB as well as its supplying triggers (cytokines, enzymes, growth factors) in CRC cells was demonstrated in various cell lines, such as HCT-116, SW480 and RKO [39], [84], [113], and is directly associated with p53-related down-regulation of cell plasticity and apoptosis induction (Table 2), because it results in insulin-like growth factor (IGF) receptor blockade leading to apoptosis [113]. Complementary, IGF receptors were inhibited by resveratrol through PTEN/PI3K/Akt/Wnt/β-catenin [114] as well as IGF/Akt/Wnt [115] pathway modulation.

In addition, an involvement of β1-integrin surface receptors with known link to inflammation-modulator NF-κB as well as neoangiogenesis-inducer HIF-1α was demonstrated in CRC cells (HCT-116, RKO, SW480) (Table 2) and an interruption of these connections led to apoptosis [39], [41]. Resveratrol’s inhibition of FAK, which is also associated with NF-κB and cancer cell plasticity, had similar effects in HCT-116 and SW480 CRC cells [116]. Furthermore, against the background of a known crosstalk between Hippo/YAP and NF-κB cascades, it is also understandable that resveratrol uses an activation of protein kinase Hippo and its downstream effector YAP to initiate apoptosis and inhibit cell phenotypic plasticity (Table 2) in CRC cells [117]. Interestingly, many authors also report a pro-apoptotic, anti-plasticity interaction between p53 and resveratrol [102], [111], [112], [115], [118], which caught our attention and which we will explore more detailed in the following.

### Modulation of p53 signaling in anti-cellular plasticity and pro-apoptosis of CRC cells by resveratrol

In healthy cells, the activation of tumor suppressor protein p53 is inhibited by binding to the murine double minute 2 gene (mdm-2) oncogene [120]. Usually, its formation and degradation by ubiquitin-mediated proteolysis are subject to a constant turnover with a short half-life [121]. But various irritations of a healthy cell, especially hypoxia [122], DNA damage [123] or oncogenic activation [124], ensure the release of mdm-2-maintained inhibition and trigger the protein kinases Ataxia Telangiectasia and Rad3 (ATR) and Ataxia Telangiectasia Mutated (ATM) [125] (Fig. 3). As a result, p53 is activated and initiates numerous molecular cascades. Some of the best known of these are the modulation of p21/cyclin D1 interaction giving the cell a chance to self-repair [126], [127] as well as promotion of Bax/cytochrome *C*/caspase-3-axis inducing cell death [128], [129]. Furthermore, the activation of pro-apoptotic genes such as PUMA, phorbol-12-myristate-13-acetate-induced protein 1 (Noxa), Bax, and BH3 interacting domain death agonist (Bid) are of great importance in cancer cells including CRC [11], [13] (Fig. 3).

The complex regulation of transcriptional activity of p53 *via* a negative feedback loop [130] can be down-regulated by resveratrol [131] with far-reaching consequences. Changes in the p53 status lead to altered efficacy of classical therapies, which is relevant because a p53 mutation can occur in up to half of CRC cases [132], [133], but most cancer cells tend to initially have high levels of p53 [130]. Moreover, in a cell affected by environmental changes such as cancer, intact and activated p53 proteins can counter-regulate and arrest the cell growth. This can be achieved, for example, by modulating the energy balance through an induction of the enzymatic ‘phosphatase and tensin homolog’ (PTEN), resulting in up-regulation of maspin protein as well as down-regulation of insulin-like growth factor (IGF)/mTOR pathway [134], [135]. Furthermore, an enhanced expression of IGF binding protein 3 (IGF-BP-3) that correlates with high p53 activation, leads to IGF receptor inhibition and thus apoptosis in CRC cells [136].

Another key capability of p53 is its initiation of a cell division arrest in different stages. On the one hand, the cell cycle can be stopped in the G1 phase by p53-initiated promotion of the p21 protein expression. This inhibits the cyclin D-CDK4/6 complex as well as the cyclin E-CDK2 complex, which usually triggers the transition from the G1 stage to the S-phase, and this regulatory p53-mechanism is proven in different CRC cell lines such as SW480 and HT-29 [137]. On the other hand, p53 is also able to prevent the formation of the cyclin B-CDK1 complex in G2 stage, before the cell’s entry into the mitotic phase, as for example shown in HCT-116 and DLD-1 CRC cells [138].

If a cell is irreparably damaged, p53 is capable of direct apoptosis initiation and, because of this central function, the protein can also exploit various signaling here. Ultimately, these pathways lead to mitochondrial apoptosis initiation *via* the caspase-9/-3 cascade through the increase in ROS, stimulation of the pro-apoptotic B-cell lymphoma 2 (Bcl-2) family including Bax, PUMA [99] and Noxa [139]. Moreover, use of Fas (cell surface death) receptors to release cytochrome *C* also flows in the same pathway, as for instance investigated in COLO-205 and HT-29 CRC cells [140] (Fig. 3). Recent clinical studies in human CRC patients showed a correlation between p53-mutation and lower 5-year survival, even in non-metastatic disease [141]. Overall, the apoptotic p53-axis is of central importance for CRC prognosis, so that researchers worldwide are working to recreate a functional p53 in p53 mutant cancers. This was already successful for CRC in a rat model and significantly improved the tumor-reducing as well as apoptosis-stimulating effect of the anti-cancer drug celecoxib [142].

Interestingly, the mentioned cascades of cell plasticity and apoptosis are inducible by the phytopharmaceutical resveratrol, whose modulative capabilities are demonstrated in Fig. 3.

While the authors of earlier studies (mainly up to 2008) attributed resveratrol’s pro-apoptotic effect to a p53-independent induction of caspase cascades, numerous more recent studies (mainly from 2009 onwards) do establish a resveratrol/p53 action link and demonstrate this in different CRC cell lines and detection methods using different resveratrol concentrations as summarized in Table 3. As early as 2005, a spectrophotometry investigation provided first evidence of resveratrol-induced p53 promotion in CRC cells in a CAM model [143]. Four years later, resveratrol’s apoptosis initiation has been successfully demonstrated to be dependent on p53 at high (200 µM) concentrations. Moreover, *via* this pathway, the phytopharmaceutical enhanced the anti-CRC effects of chemotherapeutic agent 5-FU in HCT-116 cells, whereby the proof was achieved by TUNEL staining as well as centrosome amplification [118], [144] (Table 3).

**Table 3:** Resveratrol promotes p53 expression in experimental settings with CRC cells *in vitro* and *in vivo*.

**Experiment type**	**Resveratrol treatment**	**Molecular mode of action**	**Proof of p53 expression**	**Year**	**Reference**
*In vitro/vivo*, CAM model	0.1–5 µM, 4 days	Resveratrol inhibited neovascularization, promoted p53 expression and thereby attenuated tumor growth.	Spectro-Photometry	2005	[143]
*In vitro*, HCT-116 cells	200 µM, 24 h	Resveratrol‘s anti-tumor effects were compared with the Chinese herb scuttelarin. Here, resveratrol initiated apoptosis p53-dependent *via* caspase-6 pathway and acted synergistically with 5-FU.	TUNEL staining	2009	[144]
*In vitro*, HCT-116 cells	200 µM, 24 h	Resveratrol induced apoptosis *via* caspase-6 pathway and supports 5-FU effects more in p53-presence than in p53-absence, thereby reduced tumor proliferation.	Centrosome amplification	2010	[118]
*In vitro*, HT-29 and SW480 cells	50–150 µM, 24–72 h	Resveratrol inhibited IGF-1R/Akt/Wnt signaling and promoted p53 pathway, suppressing proliferation and inducing apoptosis.	Western blot	2010	[115]
*In vitro*, HCT-116 cells	25–100 µM, 48 h	Resveratrol inhibited proliferation and induced apoptosis p53-dependent with correlated Bax:Bcl-2 ratio. In this way, resveratrol potentiated the anti-tumor effects of grape seed extract.	Nucleosomal fragmentation assay (ELISA Cell Death Detection), Western blot	2011	[145]
*In vitro*, HCT-116 cells	25–100 µM, 24–72 h	Resveratrol acted anti-proliferative and pro-apoptotic by up-regulation of p53 signaling, whereby stronger in HCT-116 than in HepG2 liver cancer cells. Additionally, its senitizing effect to the cytostatic etoposide should be mentioned.	XTT assay	2013	[146]
*In vitro*, DLD-1 and LOVO cells	1–20 µM, 24–72 h	DMU-212 (methylated derivative of resveratrol) showed up-regulation of p53 and further pro-apoptotic proteins and down-regulation of anti-apoptotic proteins. Overall, its anti-tumor activity was linked to its biotransformation catalyzed by cytochrome P450 isoenzymes.	Expression pattern	2013	[152]
*In vitro*, HCT-116 cells	40–250 µM, 24–28 h	Resveratrol led to activation of ATM kinase, thus p53 up-regulation, apoptosis induction and growth restriction.	DNA damage analysis	2015	[71]
*In vitro*, HCT-116 and HT-29 cells; *In vivo*, mouse model	12.5–400 µM, 48 h 100 mg/kg, 2 weeks	Resveratrol up-regulated p53, tumor suppressor miR-34c-KITLG, inhibited IL-6 and supported oxaliplatin-sensitivity in p53-presence. This was confirmed both *in vitro* as well as *in vivo*.	Western blot	2015	[156]
*In vitro*, colon CSCs *In vivo*, mouse model	9 µM, 24 h 0.03 % w/w, 5 days	Resveratrol inhibited Wnt/β-catenin pathway, c-Myc, cyclin D1 and promoted p53 including downstream-targets, Bax/Bcl-2 ratio, cleaved PARP. The reduction of proliferation was confirmed both *in vitro* as well as *in vivo*.	Western blot	2016	[43]
*In vitro*, Caco-2 and SW480 cells	No detailed information available as article is in Chinese.	Resveratrol decreased p-Akt and increased the expression of p53, PTEN and caspase-3, thereby limited proliferation.	Western blot	2017	[149]
*In vitro*, HCT-116 cells	3–12 µM, 24–72 h	The synthesized resveratrol analogue CS up-regulated p21, p53, Fas receptor, caspase-3/-8/-9 and cleaved PARP. Altogether, it acted cytotoxic and suppressive.	Western blot	2018	[154]
*In vitro*, DLD1 and HCT-15 cells	5–40 µM, 24–72 h	Resveratrol reduced proliferation, colony growth, cyclin D1/E2, and Bcl-2. In parallel, resveratrol increased Bax and p53, inducing apoptosis.	Computational screening	2019	[148]
*In vitro*, HCT-116, CO115 and SW48 cells	12.5–50 µM, 24 h	Resveratrol up-regulated p53, Bax, cleaved caspase-3 and PARP expression leading to initiation of apoptosis.	Western blot	2019	[147]
*In vitro*, HCT-116 cells	10–100 µM, 72 h	Akt1, IL-6, p53, VEGF, and MAPK1 were identified as resveratrol‘s central targets within the scope of its anti-cancer effects.	Biological function analysis	2019	[74]
*In vitro*, SW480 and SW620 cells	5–35 µM, 24–72 h	Resveratrol-curcumin hybrids were able to bind to p53, MMP-7 and caspase-3/-7. Overall, they acted cytotoxic and pro-apoptotic.	Molecular docking analysis	2022	[155]
*In vitro*, HT-29 cells	5–75 µg/ml, 48 h	Resveratrol derivates activated p21, p53, Bax and caspases. This confirmed them as anti-oxidative, vitality-inhibiting and anti-tumor agents.	MTS assay	2023	[153]
*In vitro*, HCT-116 cells	1–40 µM, 10–14 days	Resveratrol activated Sirt-1 at low concentrations (<5µM), but inactivated Sirt-1 at higher concentrations. In parallel, high-concentrated resveratrol (>10 µM) significantly stimulated p53 expression inducing apoptosis. By modulating TME crosstalk, resveratrol limited viability and proliferation.	Western blot, immuno-precipitation, immuno-fluorescence	2023	[75]

Per biological function analysis, the serine-threonine protein kinase Akt1, IL-6, the MAPK signaling, Vascular Endothelial Growth Factor (VEGF) and also tumor protein p53, were identified as resveratrol’s central targets in HCT-116 cells representing the most investigated CRC cell line [74]. Other authors found in the same CRC cell line, that the anti-proliferative and anti-phenotypic and metabolic plasticity acting natural polyphenol induced p53-dependent apoptosis with correlated Bax:Bcl-2 ratio from a treatment with 25 µM resveratrol on [145]. Refining this, an addition of 25 µM resveratrol showed an activation of ATM, which is required for p53 activation, in HCT-116 cells [71]. Additionally, the phytopharmaceutical acted more effectively against proliferation and for p53-related anti-plasticity/pro-apoptosis induction in these CRC cells as in HepG2 liver cancer cells at a concentration range between 25 µM and 100 µM [146] emphasizing resveratrol‘s special suitability as CRC inhibitor (Table 3). A further comparative Western blot investigation confirmed p53-up-regulation but also a Bcl-2 as well as cleaved-caspase-3 level movement towards programmed cell death in HCT-116, CO115 and SW48 CRC cells involving SET7/9 domain [147].

Resveratrol’s p53-regulation was reproduced across several CRC cell lines (Table 3). In DLD-1 and HCT-15 cells, for example, it reduced proliferation, colony growth, cell plasticity, cyclin D1/E2 as well as Bcl-2 expression with simultaneous increase of Bax and p53 [148]. Moreover, an enhancement of p53, PTEN and cleaved-caspase-3 was detected by Western blot in Caco-2 and SW480 cells [149]. The IGF-1R/Akt/Wnt signaling served as further p53-inducing pathway that could be modulated by resveratrol at 100–150 µM [115].

An exciting fact about the modulatory effect of resveratrol on inflammation can be found with regard to COX-2. Constitutive expressed COX-2 is associated with tumor promotion and resveratrol is able to reduce the production of this anti-apoptotic COX-2. But further, the grape ingredient appears to be able to specifically trigger inducible COX-2 and move it into the nucleus of a cancerous cell, where its accumulation inhibits cell plasticity *via* initiating p53 activation and thus apoptosis [150]. Furthermore, recent findings indicate resveratrol’s concentration-dependent control of p53/Sirt-1 counteract, whereby the changeover point seems to be at approximately 10 µM. In sum, low-concentrated (<5µM) resveratrol activates Sirt-1 enzyme while high-concentrated resveratrol (>10 µM) promotes apoptosis and inhibition of cell plasticity using p53 signaling pathway [75]. This confirms observations already made on other cancer cells such as Hodgkin-lymphoma cells [151].

To complete the impressions gained from *in vitro* examination, similar mechanisms of action for modified resveratrol forms are now known (Table 3). DMU-212, a methylated derivate of the natural polyphenol, showed an up-regulation of pro-apoptotic proteins including p53 and, in parallel, down-regulated the expression of anti-apoptotic proteins in DLD-1 and LOVO CRC cells [152]. Further, resveratrol derivates confirmed these effects and demonstrated a significant activation of p21, p53, Bax and caspase cascades in HT-29 cells [153]. The same pathways were triggered by a synthesized resveratrol analogue in HCT-116 cells [154] and also polyphenolic resveratrol-curcumin hybrids bound to p53, MMPs as well as caspase-3 and caspase-7 to suppress cell plasticity and induce apoptosis in SW480 and SW620 cells [155]. In the meantime, some research groups have also carried out combined *in vitro/in vivo* experiments. For example, a treatment with 50 µM resveratrol up-regulated p53 expression in HCT-116 cells while 100 µM resveratrol showed comparable results in HT-29 cells. These findings, leading to strengthened effectiveness of chemotherapeutic agent oxaliplatin, were reproduced in a mouse model [156]. Even in both, *in vitro* as well as *in vivo*, a 25 µM resveratrol supplementation promoted the activation of p53 as well as its down-stream targets by modulation of Wnt/β-catenin, cyclin D1 and Bax/Bcl-2 signaling in CRC stem cells [43].

Altogether, according to the current state of research, everything points to an inclusion of the p53-axis in resveratrol’s anti-plasticity and pro-apoptosis initiation (Table 3). The natural substance thus underlines a multi-targeting potential recommending it as a co-therapeutic for CRC, especially as it modulates the carcinogenesis in all stages (initiation, promotion, progression) and even enhances the effect of classic chemotherapeutic agents through this synergistic interaction with p53.

## Controversy about resveratrol’s Sirt-1/p53 regulation in CRC cells

To evaluate the presented influences of resveratrol on malignant plasticity in CRC cells *via* p53 pathway, the controversy existing in the literature about resveratrol’s Sirt-1/p53 modulation needs to be discussed.

Sirt-1 enzyme represents a Nicotinamide-Adenine-Dinucleotide (NAD)^+^-dependent deacetylase that is involved in regulation of different cell signaling cascades and its association with cell differentiation, metabolism, inflammation as well as cell death is a relevant subject in CRC [157], [158], [159]. A special feature is the two-sided effect of Sirt-1, because while it predominantly protects cells from carcinogenic transformation, its enzymatic activation can also deactivate apoptotic proteins and thus promote tumor cell plasticity [160]. Consequently, it is an interesting target in anti-CRC therapy and the natural substance resveratrol has demonstrated to approach Sirt-1 to exert a viability-inhibition in CRC cells [89], [159]. Resveratrol’s Sirt-1 activation leads to deacetylating protein modifications [161], which at first seems to contradict an acetylation-based p53 activation which has also been demonstrated in CRC cells [143], [146].

Over the years, a controversy has developed whose solution lies in a different pathway choice depending on the concentration of the nature-derived polyphenol. In this relation, Radhakrishnan et al. and Vanamala et al. showed a p53-initiation with corresponding Bax:Bcl-2 ratio at higher resveratrol concentrations from 25 µM to 100 µM in CRC cells, while it was not observable at lower concentrations [115], [145]. Following this approach, other and our research group recently examined resveratrol’s effects at concentrations from 1 µM to 60 µM with the finding of an interesting concentration-dependent switch in Sirt-1 or p53 interaction in the same cell line [75]. Here, low concentrations of the phytopharmaceutical (<5µM) activated the deacetylase Sirt-1 instead of p53. Nevertheless, at higher concentrations (>10 µM) of resveratrol down-regulated Sirt-1 paving the way for p53 acetylation. The higher the resveratrol concentration the stronger was p53 promotion and p53-dependent apoptosis induction as well as the inhibition of cell plasticity [75] so that a foundation has been laid for clarifying a long-standing research question. Hereby, both activations are compatible with each other, considering the resveratrol concentration used [147], [151], [162]. Moreover, apart from inconsistent standards and, cell types, nutrition mediums, culture models in different international laboratories, this offers a possible explanation for the previously debatable assessment of p53-dependent or p53-independent apoptosis promotion by the natural substance.

Overall, *in vitro* studies revealed striking chemopreventive functions through the reduction of cell stress, ROS, inflammatory processes and the inactivation of carcinogens [56], so that resveratrol appears to have modulative potential in all stages of carcinogenesis, namely initiation, promotion and progression. In this context, the modulation of p53 cascade is suggested as one of resveratrol’s key targets in different cancer types [163] and CRC cells [75]. However, simultaneously with the underlining of the significant tumor-inhibiting effect by down-regulating the cell plasticity of malignant cells, the challenge of transferring this knowledge to animal models and clinical studies is also growing.

## Clinical trials and the challenge of transferability from bench to bedside

Despite the proven, significant anti-cancer effects of resveratrol *in vitro*, not many clinical studies have been carried out on this subject and only a few have examined its potential specifically in CRC patients. An exact transfer of the concentrations used in the cell culture laboratory to human patients was not possible, because the limiting effects of metabolism and bioavailability had to be considered as reviewed in detail by Akter et al. [163]. For example, a study found that 70 % of an oral resveratrol dose is absorbed in the human body [164]. However, due to rapid metabolization in the lungs, liver and intestines, a large proportion of the active substance is excreted in the urine. During the degradation process of resveratrol, numerous metabolites such as resveratrol sulfate glucuronides, resveratrol disulfates, resveratrol 3-O-glucuronides, resveratrol 4′-O-glucuronides, resveratrol 3-O-sulfates and resveratrol 4′-O-sulfates are formed. After an oral substitution, these are detectable in healthy intestinal tissue as well as in CRC-affected tissue and in the blood plasma of humans [165]. A significant growth inhibition of CRC cells due to the treatment with sulfate- and glucuronide-metabolites has already been validated preclinically by several research groups with the assumption that it mediates resveratrol's effect strength [166], [167], [168]. Overall, this raises the possibility that resveratrol's metabolites also play a crucial role in the observed reduction in tumor growth, for example by preventing metastasis-promoting plasticity or the induction of programmed cell death. As this weighting has not yet been completed, it is currently recommended to administer resveratrol as an original substance rather than separate metabolites in clinical settings. Fortunately, it was shown that regular intake of the phytopharmaceutical nevertheless leads to accumulation, which has a disease-preventing and CRC-fighting effect [164], [165]. Moreover, an even higher detection of resveratrol residues in colorectal tissue than in circulating blood after administration per os suggests the intestinal tract as particularly susceptible to chemoprophylaxis using this grape-derived substance [165]. It should be noted that both resveratrol as well as its metabolites accumulate significantly more in the right-sided colon than in the left-sided colon [165], which could be relevant in the patient-specific therapeutic context.

A further limitation arises from the recognition of possible undesirable side effects, because while cell-toxic effects may appear beneficial at high polyphenol concentrations (>100 µM) *in vitro*, organ toxicity must be strictly avoided when used in humans. In this regard, most healthy test subjects tolerated daily doses of up to 2.5 g well, but some people may also react to high-dose intake of resveratrol with nausea, stomach pain or headaches [169]. Due to inter-individual differences, it has not yet been possible to determine an optimal dosage for all people.

In the context of clinical investigations of resveratrol’s effect on CRC patients, to date, daily dosages of 0.5 g [165] to 5 g [170] have been chosen for 8–14 days and a good tolerability has been consistently reported, regardless of whether resveratrol was administered in its pure form [165] or micronized [170]. Interestingly, taking just 0.5 g or 1 g provided anti-carcinogenic power in the gastrointestinal tract, because both resveratrol and its metabolites were subsequently detected in the gut tissue and inhibited CRC cell proliferation by 5 % [165]. Even more promising was the finding, that a dose of 5 g resveratrol also led to enrichment in the organ most frequently affected by metastases, the liver, and increased the apoptosis marker caspase-3 there by almost 40 % [170]. Altogether, these studies were carried out on patients with advanced diseases and therefore, an even stronger effect at an earlier stage of the disease would be conceivable, especially considering resveratrol’s capability to modulate tumor initiation, promotion as well as progression, summarizing all p53-involved stages of CRC development. Suitable for this, a determination of p53 as a biomarker in CRC patients is possible [171], but to our knowledge has not yet been clinically examined in combination with a resveratrol treatment.

Despite all the euphoria, it should be emphasized, that a cancer therapy will not be successful without classic drugs, especially cytostatics, and in the event of illness, resveratrol can only be used as a chemosensitizing co-therapeutic agent that alleviates side effects. A particular potential of the phytopharmaceutical represents its early, significant anti-inflammatory action, which can prevent the initiation, promotion or progression of a tumor and while most conventional drugs are mono-target specialists, resveratrol offers a unique opportunity as a multi-step as well as poly-target agent. Altogether, the natural polyphenol seems mainly suitable to act as a long-term, harmless prophylactic and, however, this requires broad-based education of the population in order to achieve a high level of compliance.

## Summary and conclusion

CRC is a major cause of human health suffering worldwide, and conventional cancer therapies are accompanied by significant side effects to the point of chemoresistance. Therefore, it is necessary to find powerful and selective cytotoxic agents for cancer to improve not only the treatment but also alleviate the side effects. Among natural compounds, polyphenols represent an important category and have always been associated with a range of restorative, cardioprotective, anti-aging, anti-inflammatory, anti-oxidant and anti-cancer effects, including anti-CRC functions. One compound in the polyphenol group is resveratrol, which is known to modulate regulatory pathways most important for inhibiting CRC development, progression, and metastasis.

This review summarizes novel relationships between anti-functional plasticity, pro-apoptosis and modulation/stabilization of tumor protein p53 signaling in CRC cell oncogenesis as one of the important mechanisms by which resveratrol prevents CRC development, based on a series of *in vivo* and *in vitro* studies and some clinical trials. The therapeutic modifications of p53 induced by resveratrol include the regulation of its gene and protein expression, as well as its post-translational modifications such as acetylation, phosphorylation, and ubiquitination, which ensure the subcellular stability of p53 and thus influence its specific down-stream signaling activation in response to stimuli, including suppression of malignant cell plasticity (EMT, CSCs, metabolism), cell cycle regulation, senescence, and apoptosis control. Despite ongoing challenges, particularly clinical ones, future research on CRC cells with their heterogeneity of cellular phenotypes is recommended, as the phytopharmaceutical represents a promising prophylactic as well as co-therapeutic agent against tumor initiation, promotion and progression in the intestinal tract.

## Funding

This research received no external funding.

## Institutional Review Board Statement

Not applicable.

## Informed Consent Statement

Not applicable.

## Compliance with Ethics Requirements

*This article does not contain any studies involving animals performed by any of the authors.*

*This article does not contain any studies involving human participants performed by any of the authors.*

## CRediT authorship contribution statement

**Aranka Brockmueller:** Conceptualization, Validation, Data curation, Writing - review & editing. **Constanze Buhrmann:** Writing – review & editing. **Amir Reza Moravejolahkami:** Writing – review & editing. **Mehdi Shakibaei:** Supervision, Visualization, design & creation of the figures, Writing – review & editing.

## Declaration of Competing Interest

*The authors declare that they have no known competing financial interests or personal relationships that could have appeared to influence the work reported in this paper.*
